# Risk Factors Related to Acute Radiation Dermatitis in Breast Cancer Patients After Radiotherapy: A Systematic Review and Meta-Analysis

**DOI:** 10.3389/fonc.2021.738851

**Published:** 2021-11-29

**Authors:** Yuxiu Xie, Qiong Wang, Ting Hu, Renwang Chen, Jue Wang, Haiyan Chang, Jing Cheng

**Affiliations:** Cancer Center, Union Hospital, Tongji Medical College, Huazhong University of Science and Technology, Wuhan, China

**Keywords:** acute radiation dermatitis, breast cancer, radiotherapy, risk factor, meta-analysis

## Abstract

**Background:**

Acute radiation dermatitis (ARD) is the most common acute response after adjuvant radiotherapy in breast cancer patients and negatively affects patients’ quality of life. Some studies have reported several risk factors that can predict breast cancer patients who are at a high risk of ARD. This study aimed to identify patient- and treatment-related risk factors associated with ARD.

**Methods:**

PubMed, Embase, Cochrane Library, China National Knowledge Infrastructure, and WanFang literature databases were searched for studies exploring the risk factors in breast cancer patients. The pooled effect sizes, relative risks (RRs), and 95% CIs were calculated using the random-effects model. Potential heterogeneity and sensitivity analyses by study design, ARD evaluation scale, and regions were also performed.

**Results:**

A total of 38 studies composed of 15,623 breast cancer patients were included in the analysis. Of the seven available patient-related risk factors, four factors were significantly associated with ARD: body mass index (BMI) ≥25 kg/m^2^ (RR = 1.11, 95% CI = 1.06–1.16, *I* ^2^ = 57.1%), large breast volume (RR = 1.02, 95% CI = 1.01–1.03, *I* ^2^ = 93.2%), smoking habits (RR = 1.70, 95% CI = 1.24–2.34, *I* ^2^ = 50.7%), and diabetes (RR = 2.24, 95% CI = 1.53–3.27, *I* ^2^ = 0%). Of the seven treatment-related risk factors, we found that hypofractionated radiotherapy reduced the risk of ARD in patients with breast cancer compared with that in conventional fractionated radiotherapy (RR = 0.28, 95% CI = 0.19–0.43, *I* ^2^ = 84.5%). Sequential boost and bolus use was significantly associated with ARD (boost, RR = 1.91, 95% CI = 1.34–2.72, *I* ^2^ = 92.5%; bolus, RR = 1.94, 95% CI = 1.82–4.76, *I* ^2^ = 23.8%). However, chemotherapy regimen (RR = 1.17, 95% CI = 0.95–1.45, *I* ^2^ = 57.2%), hormone therapy (RR = 1.35, 95% CI = 0.94–1.93, *I* ^2^ = 77.1%), trastuzumab therapy (RR = 1.56, 95% CI = 0.18–1.76, *I* ^2^ = 91.9%), and nodal irradiation (RR = 1.57, 95% CI = 0.98–2.53, *I* ^2^ = 72.5%) were not correlated with ARD. Sensitivity analysis results showed that BMI was consistently associated with ARD, while smoking, breast volume, and boost administration were associated with ARD depending on study design, country of study, and toxicity evaluation scale used. Hypofractionation was consistently shown as protective. The differences between study design, toxicity evaluation scale, and regions might explain a little of the sources of heterogeneity.

**Conclusion:**

The results of this systematic review and meta-analysis indicated that BMI ≥ 25 kg/m^2^ was a significant predictor of ARD and that hypofractionation was consistently protective. Depending on country of study, study design, and toxicity scale used, breast volume, smoking habit, diabetes, and sequential boost and bolus use were also predictive of ARD.

## 1 Introduction

Breast cancer is the most common malignancy in women (1, 2). Due to advances in earlier screening and treatment, breast cancer mortality has greatly reduced over the past few decades (2). Adjuvant radiation therapy (RT) for patients with early-stage breast cancer undergoing breast-conserving surgery or locally advanced breast cancer with positive lymph nodes undergoing modified radical mastectomy (MRM) has become the standard treatment to reduce the local recurrence and death rates of breast cancer (3, 4).

RT targets tumor cells and induces double-stranded DNA breaks, resulting in cell damage and death, as well as damage to the surrounding normal tissue (5). Due to the rapid turnover of skin tissue, the skin is particularly sensitive to the damaging effects of radiation. Acute radiation dermatitis (ARD) is one of the most common side effects, ranging from mild erythema to wet desquamation reactions; ulcers and necrosis can occur in severe cases (6). ARD may occur 2–3 weeks after the start of RT and may last up to 4 weeks after the treatment ends. ARD can cause pain/discomfort and negatively impact patients’ quality of life, increasing the incidence of depression and anxiety in patients with breast cancer (7–9). If severe ARD occurs, the RT schedule will be changed or even terminated. Therefore, exploring the risk factors of ARD is an important priority in preventing ARD and caring for patients with breast cancer undergoing RT. According to present research reports, the development or severity of ARD is affected by several risk factors, including patient-related factors (e.g., smoking, bra size, age, ethnic origin, coexisting diseases, hormonal status, tumor site, and genetic factors) and treatment factors (e.g., beam energy, total dose of radiation, treatment techniques, volume and fraction of radiation, chemotherapy, and tamoxifen therapy) (10–19). However, inconsistencies still exist between different radiotherapy centers worldwide.

Consequently, we believe that a high-quality systematic review and meta-analysis is needed to summarize currently available data to obtain an exact conclusion. This systematic review and meta-analysis aimed to evaluate the risk factors that are significantly associated with acute ARD in women with breast cancer and provide more evidence for the prevention and management of ARD.

## 2 Materials and Methods

A systematic review and meta-analysis was performed according to the Preferred Reporting Items for Systematic Reviews and Meta-Analyses (PRISMA) guidelines. The study protocol was registered in PROSPERO (https://www.crd.york.ac.uk/prospero/display_record.php?ID=CRD42021250289) (20). The PRISMA 2020 checklist is shown in **Supplementary Table 1**.

### 2.1 Search Strategy

Articles in three English databases (PubMed, Embase, and Cochrane Library) and two Chinese databases (China National Knowledge Infrastructure and WanFang databases) were searched from January 2000 to May 2021. A manual search of the reference lists of the identified literature and systematic reviews was also conducted. Only articles published in English or Chinese were included. Based on a combination of MeSH terms and keywords, the following research terms were used: “breast cancer,” “radiotherapy,” and “radiation dermatitis.” The search strategy is shown in **Supplementary Table 2**.

### 2.2 Selection Criteria

The included study needed to meet the following criteria: 1) the research participants were breast cancer patients aged 18 years and older undergoing radiotherapy; 2) the purpose of the study was to assess patients’ skin toxicity reactions and tumor- and treatment-related factors that increase the risk of radiation-induced acute skin toxicity in breast cancer patients; 3) the study outcomes were the prevalence, incidence, and severity of acute skin reactions (radiation dermatitis and erythema) induced by radiotherapy; 4) the study design was a randomized controlled trial (RCT) or observational study design, including cohort and case–control studies; and 5) relative risks (RRs), odds ratios (ORs), and hazard ratios (HRs) were used as measures of effect.

Studies were excluded if they were books, reviews, case reports, experimental laboratory articles, conference abstracts, opinion articles, commentaries, and editorial reviews.

### 2.3 Data Extraction

Study selection and data extraction were performed by two independent authors using the PRISMA flow diagram. Dissent was resolved by discussion or consultation with a third author. When data were incomplete, the original authors were contacted.

The following data were extracted for each article: first author, country, publication year, study design, study period, patient characteristics, follow-up duration, sample size of participants, all risk factors investigated, and outcome measured. Finally, the adjusted OR, RR, and HR, and 95% CIs and *p*-values were also gathered.

### 2.4 Risk of Bias Assessment

The Cochrane Handbook for Systematic Reviews was used to assess the quality of RCTs. Quality scoring of the observational study was performed using the Newcastle–Ottawa Scale. Funnel plots and Egger’s tests were performed to assess publication bias, in which Egger’s regression test (21) was performed where the number of included studies was 10 or more (22).

### 2.5 Strategy for Data Synthesis

To determine the risk factors associated with ARD, the Stata version 16 software was used for data synthesis. The RR and 95% CI of the outcome variables were calculated. The pooled effect sizes of the studies were visualized using a forest plot. The random-effects model was applied to calculate the pooled RR and its 95% CI if significant heterogeneity among studies was found. Otherwise, a fixed-effects model was used. Sensitivity analysis of the study design (RCT and cohort), ARD assessment scale (Radiation Therapy Oncology Group (RTOG) and Common Terminology Criteria for Adverse Events (CTCAE)), and area (Europe, North America, Asia, and Africa) was performed. Cochran’s Q test and *I* ^2^ statistics were conducted to assess the potential heterogeneity between individual studies, with values of the latter above 75% being regarded as indicating high levels of heterogeneity. A sensitivity analysis was also carried out using the “leave-one-out” method.

## 3 Results

### 3.1 Study Selection and Characteristics

**Figure 1:**
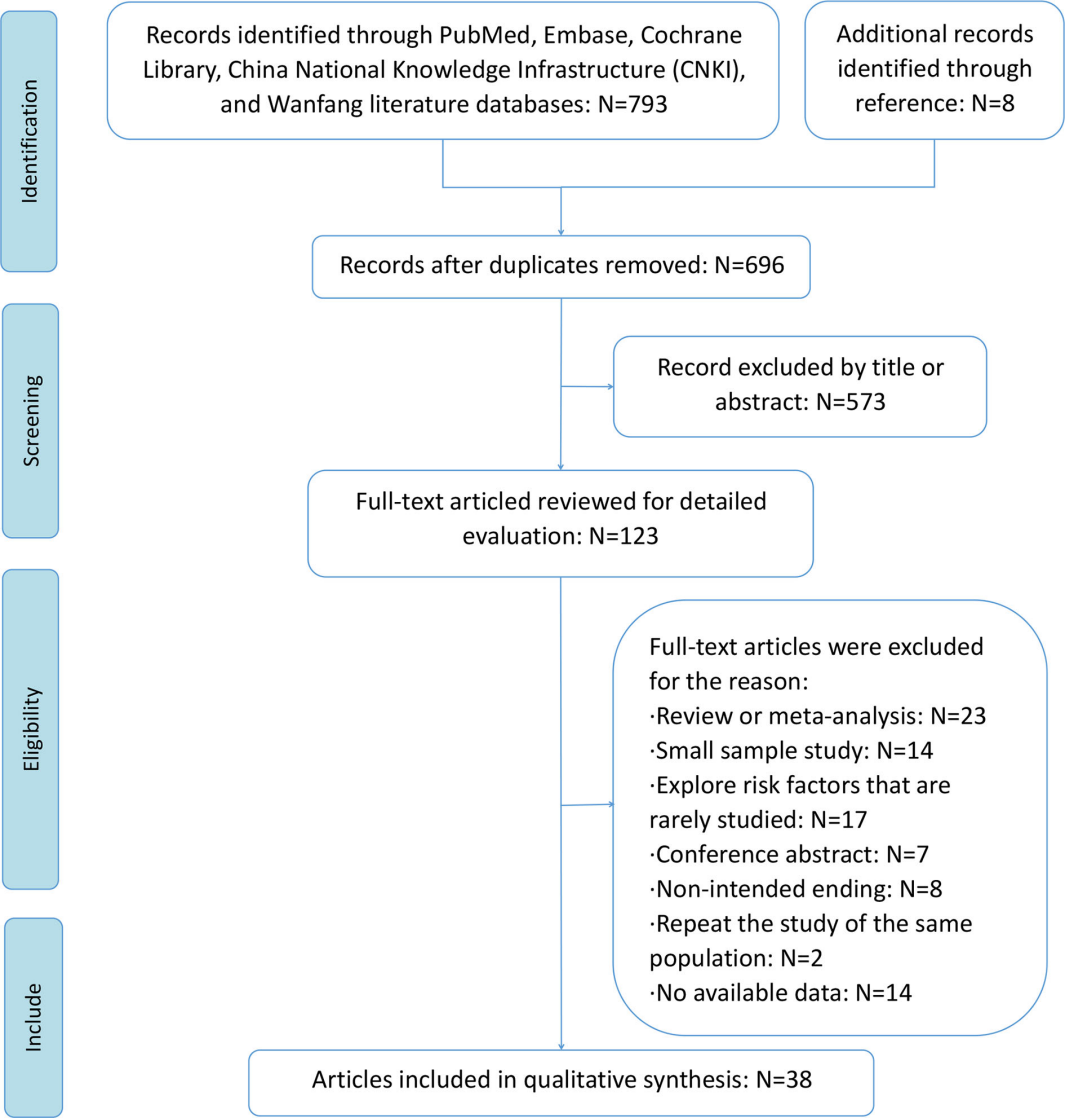
The flowchart of the literature search.

A total of 793 articles were initially identified through database searches after removing 105 duplicate articles. The abstracts and titles of 606 articles were reviewed, and 573 articles were excluded. The remaining 123 articles were read and screened in full texts for further assessment of eligibility. A total of 85 articles were further excluded for the following reasons: review or meta-analysis, small sample study, risk factors that are rarely studied, conference abstracts, non-intended endings, study being repeated on the same population, and lack of available data. Finally, 38 studies were included in this meta-analysis, of which five studies were RCTs, nine studies were retrospective designs, and 24 studies were prospective design (10–12, 14–17, 23–53). Except for one study from the WanFang database (40), all other included studies were indexed in PubMed. A flowchart of the literature search is shown in **Figure 1**.

The characteristics of the included studies are summarized in **Table 1**. The total number of patients was 15,623, ranging from 75 to 2,309 patients per study. Most patients had stage I–III breast cancer and were treated with breast-conserving surgery for early-stage breast cancer or MRM with positive lymph nodes for advanced breast cancer. None of the patients received breast reconstruction [except for some patients in the study by Aoulad et al. (36)]. The National Cancer Institute CTCAE (NCI CTCAE) and the RTOG were the most common scales used to evaluate ARD (**Supplementary Table 3**). Most centers used three-dimensional conformal radiotherapy (n = 18) or intensity-modulated RT (n = 13) technique for radiotherapy. The dose and fractionation schedule of radiotherapy used in all studies was conventional fractionated radiotherapy (CFRT), defined as a total of 50 Gy in 25 fractions over 5 weeks, or hypofractionated radiotherapy (HFRT), defined as a total dose ranging from 40 to 45.05 Gy, with a single dose of 2.3–2.9 Gy given over 13–17 fractions. The Newcastle–Ottawa Scale is shown in **Supplementary Table 4**.

**Table 1:** The characteristic of included studies.

Study ID	Study design	Country	Evaluation criterion	Total patients	The proportion of ARD with ≥2 Grade	Period	Age (range, years)	RT technique	RT dose, F and time	Boost	Risk factors
Pasquier, D.2021 (10)	PS	France	CTCAE v4.0	288	36.8%	NA	55 (32–82)	IMRT	50 Gy/25 F/5 w	Yes	Smoking; chemotherapy
Joseph, K.2021 (23)	RCT	Canada	CTCAE v3.0	177	FiF-IMRT: 61%; HT-IMRT: 37%	2008–2012	58 (41–82)	FiF-IMRT; HT-IMRT	50 Gy/25 F/5 w	No	Breast volume; chemotherapy; hormone treatment
Abdeltawab, A. A.2021 (11)	PS	Egypt	RTOG/EORTC	75	16%	2015–2018	59.47 (44–80)	2D-RT	50 Gy/25 F/5 w	Yes	Using of trastuzumab; boost
Zygogianni, A.2020 (24)	RS	Greece	RTOG/EORTC	134	NA	2004–2012	75	HF RT	Group A: 42.75 Gy/15 F/5 w; group B: 45.05 Gy/17 F	Yes	Age; treatment group: two hypofractionated radiation schedules
Rattay, T.2020 (12)	PS	UK	RTOG/CTCAE	2285	LeN: 27.1%, ISE: 74.9%, Cam: 38.9%	LeN: 2008–2010, ISE: 1998–2001, Cam2003–2007	LeN: 59, ISE: 61, Cam: 59	3D-CRT; IMRT	LeN: 50 Gy/25 F, ISE: 50 Gy/25 F, Cam: 40 Gy/15 F	Yes	BMI; breast size; HFRT; boost; smoking
Chen, C. H.2020 (14)	RS	China	RTOG	308	17.3%	2012–2018	54 (24–88)	3D-CRT; IMRT	50 Gy/25 F; 42.56 Gy/16 F	NA	Surgery type; nodal irradiation; BMI; RT technique: IMRT vs. 3D-CRT
Wang, S. L.2019 (15)	RCT	China	CTCAE v3.0	810	CFRT: 8%; HRT: 3%	2008–2016	49 (24–74)	2D-RT, 3D-CRT, IMRT	CFRT: 50 Gy/25 F/5 w; HFRT: 43.5 Gy/15 F/3 w	Yes	Treatment group: CFRT vs. HFRT
Pasquier, D.2019 (16)	PS	France	CTCAE v4.0	114	42%	2014–2016	56 (32–83)	NA	CFRT: 50 Gy/25 F/5 w	Yes	BMI; chemotherapy
Palumbo, I.2019 (25)	PS	Italy	CTCAE v4.03	219	NA	2014–2015	62 (34–88)	WBRT	HFRT: 42.4 Gy/16 F	Yes	Boost; chemotherapy
Kawaguchi, H.2019 (26)	PS	Japan	CTCAE v3.0.	348	HF-WBI: 13.8%; CF-WBI: 29.4%	2009–2013	58 (26–81)	CF-WBI; HF-WBI	CF-WBI: 50 Gy/25 F; HF-WBI: 41.6 Gy/16 F	Yes	CF-WBI vs. HF-WBI; chemotherapy; hormone treatment
Butler-Xu, Y. S.2019 (17)	RS	USA	RTOG	114	CFRT: 76%, HFRT: 28%	2012–2015	NA	3D-CRT	HFRT: 40.05 Gy/15 F; CFRT: 50 Gy/25 F/5 w	Yes	Boost; CFRT vs. HFRT
Yap, M. L.2018 (27)	PS	Canada	NA	314	16.60%	2004–2009	53.2 (27–86)	3D CRT; IMRT	50 Gy/25 F/5 w	Yes	Bolus
Rastogi, K.2018 (28)	PS	India	RTOG	100	NA	NA	48 (21–79)	3D-CRT	CFRT: 50 Gy/25 F/5 w; HFRT: 42.72 Gy/16 F/3-3.5 w	No	Treatment group: HFRT vs. CFRT
Parekh, A.2018 (29)	RS	India	CTCAE	280	31.40%	2008–2015	60	3D-CRT	CFRT, HFRT	No	Black race; BMI; treatment group: HFRT; chemotherapy; regional nodal irradiation
Lin, J. C.2018 (30)	RS	China	CTCAE v3.0	458	IMRT: 26.80%, IGRT: 14.10%	2012–2014	20–85	TOMO, IMRT	50 Gy/25 F/5 w	Yes	Age; treatment group: IGRT vs. IMRT; smoking
Guttmann, D. M.2018 (31)	RS	USA	CTCAE v4.03	413	NA	2011–2015	56	3D planning or IMRT	CFRT: 50–50.4 Gy/25 F/5 F. HFRT: 4,256 cGy/266 cGy daily	Yes	IMRT vs. FiF3D; treatment group: HFRT vs. CFRT; boost
De Santis, M. C.2018 (33)	PS	Italy	RTOG	727	21.9%–28.4%	2009–2016	74 (47–92)	Hypo-RT	NA	Yes	Chemotherapy; boost; trastuzumab
Das, Pabitra.2018 (34)	RCT	India	RTOG	108	CFRT: 24.5%; HFRT: 23.6%	2013–2015	49	2D-RT	CFRT: 50 Gy/25 F/5 w; HFRT: 42.56 Gy/16 F/3.1 w	No	Treatment group: HFRT vs. CFRT
Fatma M. F.2018 (32)	RCT	Egypt	RTOG	100	HFRT: 16%, CFRT: 26%	2015–2017	31–68	3D-CRT	CFRT: 50 Gy/25 F/5 w; HFRT: 40 Gy/15 F/3 w	Yes	Treatment group: HFRT vs. CFRT
De Felice, F.2017 (35)	PS	Italy	CTCAE v4.0	120	HFRT: 26.5%, CFRT: 73.5%	2012–2015	58 (39–82)	NA	CFRT: 50 Gy/2 Gy daily; HFRT: 42.5 Gy/2.66 Gy daily	Yes	Chemotherapy
Aoulad, N.2017 (36)	RS	France	CTCAE v4.0	292	24.6%	2010–2014	NA	IMRT	Conservative surgery: 52.2-63.8 Gy/29 F; mastectomy: 50 Gy/25 F	NA	BMI
Wright, J. L.2016 (37)	PS	USA	CTCAE v3.0	392	52%	2008–2014	56.2 (27–85)	Field-in-field technique	CFRT: 50 Gy/2 Gy daily; HFRT: 42.4 Gy/2.65 Gy daily	Yes	Age; race; BMI; treatment group: CRT vs. HFRT; breast volume.
Linares, I.2016 (38)	PS	Spain	CTCAE v4.0	143	9.8%	2006–2011	73 (50–86)	3D-CRT	HFRT: 42.4 Gy/16 F/2.65 Gy daily	Yes	RT volume; simultaneous boost (SIB) vs. none; boost: not simultaneous boost vs. none
Córdoba, E. E.2016 (39)	PS	USA	RTOG	80	40%	NA	59 (26–79)	3D-CRT	CFRT: 50–50.4 Gy/1.8–2 Gy daily	Yes	BMI; breast size
Zhang, S. K.2015 (40)	PS	China	CTCAE v4.03	786	12.9%	2009–2014	NA	3D-CRT	50 Gy/25 F/5 w	NA	Diabetes; BMI; neoadjuvant chemotherapy
Pignol, J. P.2015 (41)	PS	Canada	CTCAE v3.0	257	28.4%	2005–2007	51 (24–80)	Photon beams or direct electron field and photon tangent fields	50 Gy/25 F/5 w	Yes	Smoking; chemotherapy; bolus frequency
Jagsi, R.2015 (42)	PS	USA	CTCAE v4.0	2309	CFRT: 62.6%; HFRT: 27.4%	2011–2014	61.2	NA	NA	Yes	Treatment group: CFRT vs. HFRT
Wright, J. L.2014 (43)	PS	USA	CTCAE v3.0	110	NA	2010–2013	51.9 (28–75)	NA	50 Gy/25 F/5 w	NA	Age; ethnicity; race; BMI; smoking; chemotherapy
Park, H.2014 (44)	PS	Korea	RTOG	213	27%	NA	42 (21–71)	NA	50–65 Gy/1.8–2 Gy daily	Yes	Age; BMI; breast volume; diabetes; hypertension; chemotherapy; hormone therapy
De Langhe, S.2014 (45)	PS	Belgium	CTCAE v3.0	377	58%	NA	58 (30–82)	IMRT	CFRT: 50 Gy/25 F/5 w; HFRT: 40 Gy/15 F/3 w	Yes	BMI; breast size; smoking; HFRT vs. CFRT; hormone therapy; chemotherapy; trastuzumab
Ciammella, P.2014 (46)	PS	Italy	RTOG	212	15%	2009–2012	63 (39–88)	3D-CRT	HFRT: 40.05/15 F/2.67 Gy daily	Yes	Breast volume; boost
Tortorelli, G.2013 (47)	RS	Italy	RTOG	339	CFRT: 55%; HFRT: 37.5%	2007–2010	60 (22–86)	3D-CRT	CFRT: 50 Gy/25 F/5 w; HFRT: 44 Gy/16 F/2.75 Gy daily	Yes	Chemotherapy; hormone therapy; fractionation schedule; age; breast volume
Sharp, L.2013 (48)	PS	Sweden	RTOG/EORTC	390	21%	2010–2011	59 (29–86)	NA	50 Gy/2.0 Gy daily; 42.56 Gy/2.66 Gy daily	Yes	Age; BMI; smoking; surgery: chemotherapy; endocrine therapy
Terrazzino, S.2012 (49)	PS	Italy	RTOG	286	31.1%	2009–2011	60.8	3D-CRT	CFRT: 50 Gy/25 F/5 w	Yes	Breast size; boost; BMI
Freedman, G. M.2009 (51)	RS	USA	CTCAE v3.0	804	Conventional: 75%, IMRT: 52%	2001–2006	NA	Wedged photon tangents and IMRT	46–50 Gy	Yes	CRT vs. IMRT; breast size; chemotherapy; hormone therapy
Morganti, A. G.2009 (50)	PS	Italy	NA	332	CG: 33.6%, MARA-1: 13.1%, MARA-2: 45.1%	NA	57.5	3D-CRT	MARA-1: HFRT; 40 Gy/2.5 Gy daily; MARA-2: CFRT: 50 Gy/2 Gy daily	Yes	HFRT vs. CFRT; hypertension; diabetes; smoke; hemoglobin; age; hormone therapy; chemotherapy
Pignol, J. P.2008 (52)	RCT	Canada	CTCAE v2.0	331	IMRT: 31.2%, standard treatment: 47.8%	2003–2005	57	Standard wedge missing tissue or IMRT	50 Gy/25 F/5 w	Yes	Treatment group: BIMRT technique Breast size; boost
Back, M.2004 (53)	PS	Germany	CTCAE	478	17.5%	1998–2001	NA	NA	50 Gy/2 Gy/F; 50.4 Gy/1.8 Gy daily	Yes	Radiotherapy of lymph nodes; hormone therapy; age; BMI; smoking

PS, prospective study; RS, retrospective study; RCT, randomized controlled trial; HFRT, hypofractionated radiotherapy; CFRT, conventional fractionated radiotherapy; FiF-IMRT; field-in-field intensity-modulated radiotherapy; HT-IMRT; helical tomotherapy intensity-modulated radiotherapy; CTCAE, Common Terminology Criteria for Adverse Events; RTOG, Radiation Therapy Oncology Group; F, fractions; w, weeks; 2D-RT, two-dimensional radiotherapy; 3D-CRT, three-dimensional conformal radiotherapy; IMRT, intensity-modulated radio therapy; AA, African-American; BMI, body mass index; LeN, LeND cohort; ISE, ISE cohort; Cam, Cambridge cohort; EORTC, European Organisation for Research and Treatment of Cancer; WBRT, whole brain radiotherapy; HF-WBI, hypofractionated whole-breast irradiation; CF-WBI, conventionally fractionated whole-breast irradiation; IGRT, image-guided radiation therapy; TOMO, tomotherapy.

### 3.2 Meta-Analysis of Risk Factors

#### 3.2.1 Patient-Related Risk Factors

**Figure 2:**
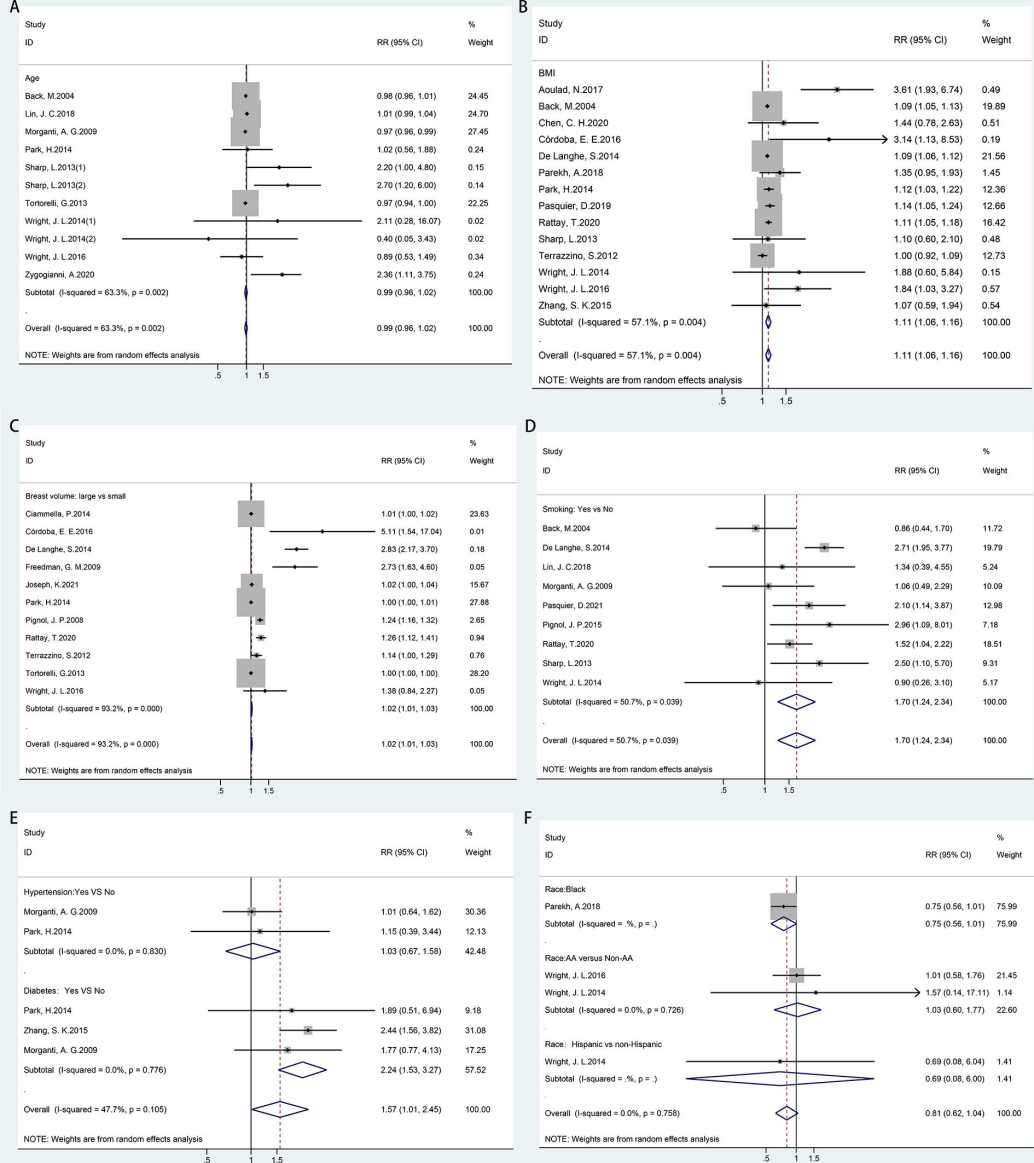
Forest plot of studies among patient-related risk factors associated with acute radiation dermatitis. **(A)** Age. **(B)** Body mass index (BMI). **(C)** Breast volume. **(D)** Smoking. **(E)** Chronic disease (hypertension/diabetes). **(F)** Race.

Random-effects meta-analyses were conducted for patient-related risk factors, including age, body mass index (BMI), breast volume, smoking, race, hypertension, and diabetes, as shown in **Figure 2**. It was found that patients with BMI ≥ 25 kg/m^2^ (RR = 1.11, 95% CI = 1.06–1.16), large breast volume (RR = 1.02, 95% CI = 1.01–1.03), smoking habits (RR = 1.70, 95% CI = 1.24–2.34), or diabetes (RR = 2.24, 95% CI = 1.53–3.27) had significantly higher risks of ARD than their counterparts. However, a significantly increased risk was not observed with increasing age (RR = 0.99, 95% CI = 0.96–1.02), hypertension (RR = 1.03, 95% CI = 0.67–1.58), or race (RR = 0.81, 95% CI = 0.62–1.04).

According to Cochran’s Q test and *I*^2^ statistics, a substantially large inconsistency (*p* = 0.000, *I* ^2^ = 93.2%) was found for significant heterogeneity among studies regarding the breast volume. BMI (*p* = 0.004, *I* ^2^ = 57.1%) and smoking habits (*p* = 0.039, *I* ^2^ = 50.7%) showed moderate inconsistency with significant heterogeneity in each meta-analysis. No heterogeneity was found for hypertension, diabetes, or race. There was no indication of publication bias, as implied by the funnel plot and Egger’s tests for the risk factors of age (*p* = 0.084) and smoking habits (*p* = 0.284). However, funnel plots and Egger’s test indicated potential publication bias for BMI (*p* = 0.016) and breast volume (*p* = 0.001) (**Supplementary Figure 1**).

#### 3.2.2 Treatment-Related Risk Factors

**Figure 3:**
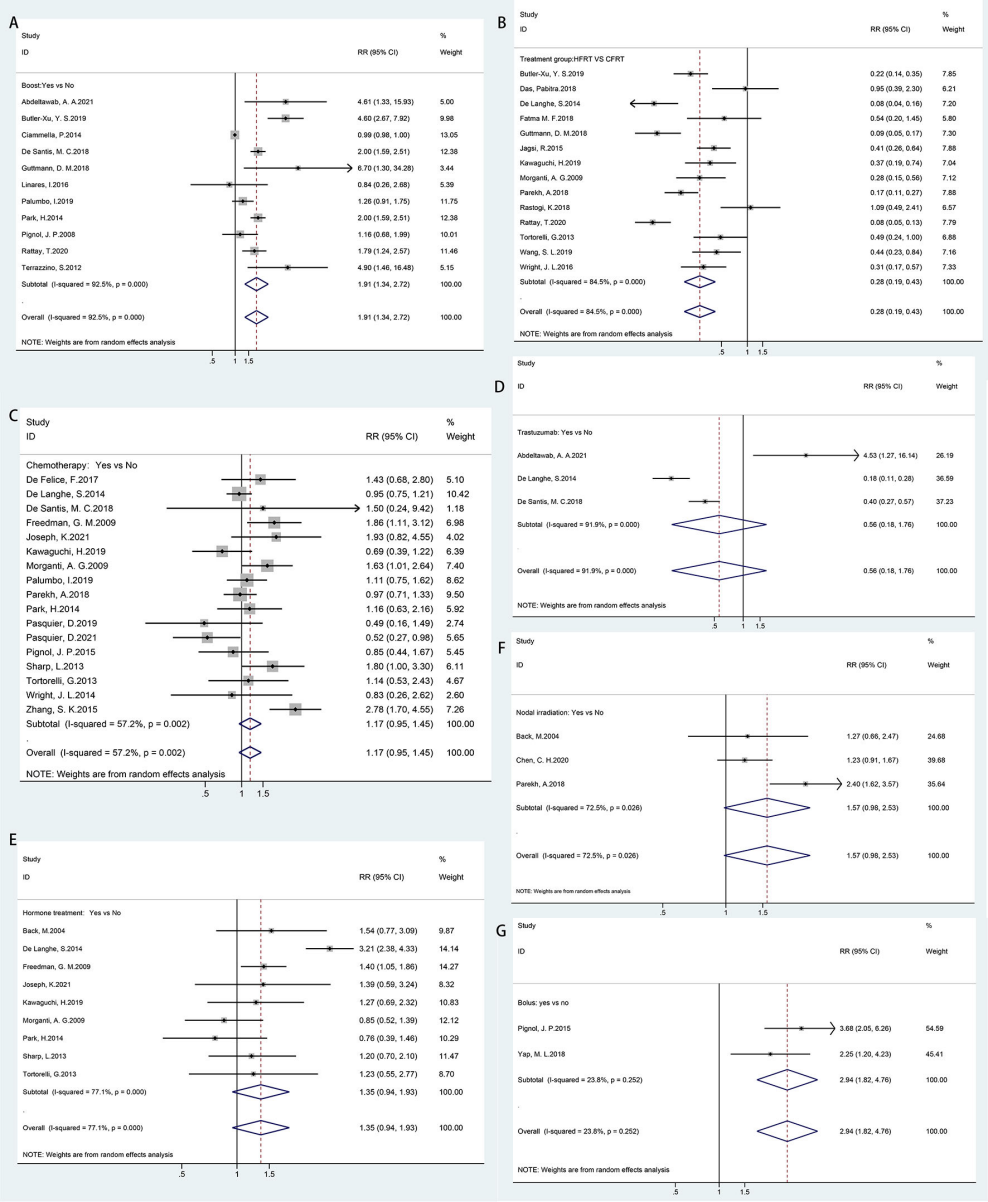
Forest plot of studies among treatment-related risk factors associated with acute radiation dermatitis. **(A)** Boost. **(B)** Hypofractionated radiotherapy (HFRT) vs. conventional fractionated radiotherapy (CFRT). **(C)** Chemotherapy regimen. **(D)** Trastuzumab therapy. **(E)** Hormone therapy. **(F)** Nodal irradiation. **(G)** Bolus.

In **Figure 3**, all treatment-related risk factors available for meta-analyses were performed on sequential boost (boost vs. non-boost), chemotherapy regimen (yes vs. no), hormone therapy (yes vs. no), trastuzumab therapy (yes vs. no), HFRT versus CFRT, bolus (yes vs. no), and nodal irradiation (yes vs. no). Our results indicated that HFRT reduced the risk of ARD in breast cancer patients as compared with CFRT (RR = 0.28, 95% CI = 0.19–0.43). Sequential boost and bolus use was significantly associated with ARD (boost, RR = 1.91, 95% CI = 1.34–2.72; bolus, RR = 1.94, 95% CI = 1.82–4.76). However, chemotherapy regimen (RR = 1.17, 95% CI = 0.95–1.45), hormone therapy (RR = 1.35, 95% CI = 0.94–1.93), trastuzumab therapy (RR = 1.56, 95% CI = 0.18–1.76), and nodal irradiation (RR = 1.57, 95% CI = 0.98–2.53) were not correlated with ARD.

Considerable heterogeneity was observed among the risk estimates for chemotherapy (*p* = 0.002, *I* ^2^ = 57.2%), sequential boost (*p* = 0.000, *I* ^2^ = 92.5%), hormone therapy (*p* = 0.000, *I* ^2^ = 77.1%), HFRT versus CFRT (*p* = 0.000, *I* ^2^ = 84.5%), trastuzumab therapy (*p* = 0.000, *I* ^2^ = 91.9%), and nodal irradiation (*p* = 0.026, *I* ^2^ = 72.5%). No statistically significant heterogeneity was detected for bolus (*p* = 0.252, *I* ^2^ = 23.8%). No evidence of asymmetry in the funnel plot was found, and Egger’s tests also showed no significant evidence of publication bias for chemotherapy (*p* = 0.676), hormone therapy (*p* = 0.152), or HFRT versus CFRT (*p* = 0.07). However, a potential publication bias was observed for boosts in the funnel plot and Egger’s test (*p* = 0.002) (**Supplementary Figure 2**).

### 3.3 Sensitivity Analysis

Sensitivity analysis by study design, ARD assessment scale, and regions was performed on the risk factors that more than 10 studies reported and included. As shown in **Table 2**, in each subgroup, the results of the risk factors (age, BMI, chemotherapy, and HFRT vs. CFRT) did not change significantly; however, the results of heterogeneity were slightly improved. BMI was consistently associated with ARD. Hypofractionation is consistently shown as a protective factor. The results of studies from European countries showed that smoking was a risk factor for ARD; however, studies in North America and Asia indicated that smoking was not associated with ARD. The combined results of three studies from North American countries also suggested that a boost was not related to ARD. For the two risk factors of breast volume and boost, the RCT results suggested no statistical significance, but the results of the prospective cohort study were significant. These contradictory results may be because only one or two related RCT studies were included in the meta-analysis, but such results also proved the heterogeneous results of the study design. In addition, our results showed that when the CTCAE was used to assess ARD, large breast volume increased the risk, but an irrelevant association was observed when the RTOG criteria were used. Inconsistent results were also observed between the boost and ARD according to the different assessment criteria. The difference between the CTCAE scale (which incorporates inframammary desquamation in grade 2) and RTOG criteria may explain a little of the sources of heterogeneity. Besides, after removing one study every time, the significance of the results remained consistent, which indicated that our results were stable (**Supplementary Figures 3**, **4**).

**Table 2:** Sensitivity analysis by study design, acute radiation dermatitis evaluation scale and area.

Factors	All	Area				Study design			Evaluation scale	
		Europe	North America	Asia	Africa	Prospective study	Retrospective study	RCT	RTOG	CTCAE
	RR (95% CI)	RR (95% CI)	RR (95% CI)	RR (95% CI)	RR (95% CI)	RR (95% CI)	RR (95% CI)	RR (95% CI)	RR (95% CI)	RR (95% CI)
Age	0.99 (0.96–1.02)	0.98 (0.94–1.02)	0.99 (0.55–1.46)	1.01 (0.99–1.04)		0.98 (0.94–1.01)	1.00 (0.94–1.07)		1.57 (0.97–2.57)	1.00 (0.98–1.01)
BMI	1.11 (1.06–1.16)	1.09 (1.05–1.14)	2.06 (1.30–3.26)	1.13 (1.04–1.23)		1.09 (1.06–1.13)	1.85 (1.03–3.31)		1.09 (1.01–1.18)	1.13 (1.06–1.20)
Smoking	1.70 (1.24–2.34)	1.71 (1.18–2.47)	1.73 (0.54–5.53)	1.34 (0.39–4.58)		1.72 (1.23–2.41)	1.34 (0.39–4.58)		1.70 (1.13–2.54)	1.75 (1.11–2.78)
Breast volume	1.02 (1.01–1.03)	1.05 (1.01–1.09)	1.32 (1.08–1.62)	1.00 (1.00–1.01)		1.06 (1.02–1.10)	1.60 (0.60–4.26)	1.12 (0.93–1.35)	1.00 (1.00–1.01)	1.55 (1.22–1.97)
Chemotherapy	1.17 (0.95–1.45)	1.10 (0.85–1.42)	1.35 (0.85–2.14)	1.22 (0.69–2.14)		1.12 (0.86–1.46)	1.24 (0.80–1.91)	1.93 (0.82–4.55)	1.38 (0.96–1.99)	1.09 (0.84–1.42)
Hormone treatment	1.35 (0.94–1.93)	1.47 (0.81–2.69)	1.40 (1.07–1.83)	1.00 (0.60–1.65)		1.33 (0.77–2.29)	1.38 (1.05–1.81)	1.39 (0.59–3.26)	1.04 (0.72–1.51)	1.72 (1.09–2.71)
Boost	1.91 (1.34–2.72)	1.51 (1.02–2.24)	2.94 (0.94–9.14)	2.00 (1.59–2.51)	4.61 (1.33–15.95)	1.70 (1.17–2.47)	4.78 (2.85–8.00)	1.16 (0.68–1.99)	2.24 (1.41–3.57)	1.30 (0.86–1.96)
HFRT vs. CFRT	0.28 (0.19–0.43)	0.17 (0.07–0.40)	0.23 (0.12–0.42)	0.47 (0.23–0.96)	0.54 (0.20–1.45)	0.26 (0.13–0.51)	0.20 (0.11–0.35)	0.57 (0.36–0.90)	0.39 (0.17–0.93)	0.22 (0.13–0.37)

RCT, randomized controlled trial; HFRT, hypofractionated radiotherapy; CFRT, conventional fractionated radiotherapy; CTCAE, Common Terminology Criteria for Adverse Events; RTOG, Radiation Therapy Oncology Group; BMI, body mass index; RR, relative risk.

## 4 Discussion

The proportion of patients with ARD of grade 2 or higher after radiotherapy ranged from 9.8% to 76%, with an average of 34.3% and a median of 28.4%, across the 38 included studies. This study aimed to identify the risk factors associated with ARD so that clinicians could assess the risk of toxicity at the time of breast cancer diagnosis and before planning any treatment, as well as adjust treatment decisions and take preventive measures in advance. Our results indicated that several variables, including BMI, breast volume, smoking habits, diabetes, boost and bolus use, and hypofractionation (protective), were related to ARD. Age, hypertension, chemotherapy, hormone therapy, trastuzumab therapy, and nodal irradiation were not associated with radiation dermatitis.

The 10-year follow-up of the UK Standardisation of Breast Radiotherapy (START) trials confirmed that appropriately dosed HFRT was safe and effective in patients with early breast cancer (54–57). Normal tissue effects (breast induration, shrinkage, telangiectasia, and breast edema) were significantly less common in the HFRT group than that in the CFRT group (54, 55). Another randomized, non-inferiority, phase 3 trial reported that the HFRT (43.5 Gy over 15 fractions in 3 weeks) and CFRT groups (50 Gy over 25 fractions in 5 weeks) had equivalent efficacy in the 5-year locoregional recurrence, overall survival, and disease-free survival in patients with high-risk breast cancer (15). This trial did not find a significant difference in the incidence of acute or late toxicities, but there were fewer patients who experienced grade 3 acute skin toxicity in the HFRT group than that in the CFRT group (14 [3%] of 401 patients vs. 32 [8%] of 409 patients, *p* < 0.0001) (15). A meta-analysis based on large randomized trials also indicated that HFRT and CFRT were equally effective with respect to overall survival, disease-free survival, locoregional recurrence, and distant metastasis after breast MRM and had similar toxic side effects (58). Another meta-analysis concluded that no difference was found between CFRT and HFRT in terms of efficacy; however, HFRT showed a lower incidence of breast edema, telangiectasia, and acute skin radiation toxicity compared with that in CFRT (59). A large multicenter cohort found that HFRT not only improved the convenience of patients but also reduced acute pain, fatigue, and dermatitis in patients with breast cancer (42). Consistent with these studies, our results also suggest that HFRT could reduce the risk of radiation dermatitis compared with that in CFRT. Recruitment bias cannot be eliminated in nonrandomized trials, such as the hypofractionation proposed for smaller breast volumes. Therefore, we conducted a subgroup analysis according to the study design, and the results showed that HFRT could reduce the risk of ARD compared with that in CFRT according to three randomized trials. In addition, studies from the United States and Asia have reported that HFRT could reduce the treatment cost of patients by approximately 1/3 (60, 61). HFRT not only reduces the occurrence of ARD but also helps shorten the treatment cycle, reduce the length of hospital stay, save medical resources, and mitigate financial pressure, especially in low- and middle-income countries. Therefore, the National Institute for Health and Care Excellence, England’s Health Technology Assessment agency, recommends HFRT as a standard practice in patients with early-stage breast cancer who underwent breast-conserving surgery or MRM (62).

To improve the local control rate, it is necessary to administer a localized dose escalation (boost) to the tumor bed. However, as our results show, sequential application of a boost increases the risk of ARD. Therefore, it is urgent to find a suitable boost administration method that reduces the risk of side effects without compromising local control. A study found that patients with simultaneous integrated boost had lower toxicity than those receiving a sequential boost or no boost (38). Two reviews concluded that simultaneous integrated boost is a feasible approach with acceptable risk and severity of adverse events (63, 64). The Phase III trial (RTOG 1005 trial) of the North American Radiotherapy Oncology Group is currently in clinical trials, which compare the therapeutic and side effects of hypofractionated whole breast RT with a concurrent tumor bed boost versus standard daily RT with a sequential boost. We look forward to the results of the trial, which could improve the acceptance, shorten the overall treatment time, and broaden the applicability of HFRT in patients with breast cancer (65).

Our results showed that nodal irradiation was not associated with ARD, but nodal irradiation resulted in a larger irradiated volume. Regarding the dose distribution of the target volume and skin and the occurrence of skin toxicity, existing data are sparse. One prospective study found that subclavian skin volume is correlated with medium-term skin toxicity (16). Two other authors stated that dose inhomogeneities within the target volume have a significant impact on the incidence of skin reactions (47, 66). As hot spots often occur close to the skin, a more homogenous dose distribution will result in a lower incidence of skin toxicity. Therefore, it is suggested that treatment planning techniques with a more homogenous dose distribution, such as intensity-modulated RT, are shown to result in lower rates of severe skin toxicity (51, 67).

Patient-related risk factors, such as BMI, breast volume, smoking habits, and diabetes, were found to be risk factors for ARD. Large breast volume and high BMI have been most frequently reported to increase the risk of ARD. BMI is strongly related to breast volume (68). Breast volume has been used as a surrogate indicator of radiotherapy dose inhomogeneity, which may be one of the reasons for the increased ARD. However, two randomized clinical trials have highlighted breast volume as a stand-alone predictor of ARD independent of dose inhomogeneity (52, 69). It is necessary to consider that the association between a larger breast volume and the risk of ARD is likely due to the abrasive effect of friction within skin folds and the bolus effect in the inframammary, skin folds, and axillary regions. In fact, it is difficult for obese people to lose weight in a short period of time. In addition, weight changes during RT planning and radiotherapy will obviously change the treatment area; hence, weight loss is not recommended at this point. We recommend that breast cancer patients keep their skin dry and avoid friction at the skin folds during radiotherapy. Smoking increases the risk of radiation dermatitis; therefore, quitting smoking during radiotherapy is one of the best decisions that patients can make to reduce the risk of ARD. It was found that diabetic patients have a higher risk of ARD, but this result was obtained from three prospective studies only. Whether diabetes is related to radiation dermatitis and the repair mechanism of radiation damage caused by abnormal metabolism in the skin requires further research.

It was necessary to investigate potential sources of heterogeneity. First, the type of research design included in the study is different, resulting in substantial heterogeneity. Therefore, a sensitivity analysis was conducted according to the type of study (prospective cohort study, retrospective cohort, and RCT). For the two risk factors of breast volume and boost, the results of the prospective cohort study and RCT were conflicting, proving the heterogeneous results of the study design. Second, acute toxicity was evaluated using the most common tools: the NCI CTCAE or RTOG scale. One study found a high concordance between the RTOG and CTCAE criteria (correlation coefficients >0.9) (70). Nevertheless, differences still exist between the two assessment tools, such as the CTCAE scale incorporating inframammary desquamation in grade 2, leading to inconsistent assessment results of radiation dermatitis. The sensitivity analysis found that large breast volume increased the risk by the CTCAE scale, but an irrelevant association was observed when the RTOG criteria were used. Inconsistent results were also observed between the boost and ARD according to the different assessment criteria. In addition, skin toxicity was assessed at different time points, such as at the completion of the last RT, 2 weeks after the end of RT, or when the toxicity was the most serious, which caused differences in outcome reporting. Third, the use of different radiotherapy techniques inevitably caused partial heterogeneity. Several studies have demonstrated that, compared with 3D and 2D conformal radiotherapies, intensity-modulated RT provides better dose homogeneity with lower volumes of OAR receiving high doses and reduced acute and late breast toxicity (16, 52, 71, 72). Finally, heterogeneity may be partly due to ethnic differences. Similar to the sensitivity of radiotherapy, the tissue actions produced by radiotherapy are complex processes involving multiple genes in multiple biological pathways (73–76). One study reported that African-American patients with breast cancer were more likely to suffer from skin toxicity (77).

In addition to the large heterogeneity of our results, there are other shortcomings that need to be considered. First, the number of included studies was limited, especially for some risk factors (diabetes, hypertension, trastuzumab therapy, and bolus users); therefore, it is insufficient for statistical analyses, and the results should be interpreted with caution. Second, cohort studies are the main part of the included studies and carry inevitable inherent biases. Cohort studies do not use randomization; therefore, the groups may not be comparable, leading to selection bias. In addition, not all studies adjusted for confounding factors and recall bias due to selective reports, or the presentation of incomplete result data may affect the results of the analysis. These are potential deviations that may affect the validity of the research results. Third, this article only studied the risk factors of ARD; other side effects of radiotherapy, such as late radiation dermatitis, radiation pneumonitis, and radiation esophagitis, still need to be further explored. Finally, studies have reported that genetic analysis can predict patients who are at a higher risk of ARD (75, 76). However, they were single-center, small-sample studies, and each study focused on different genes. Therefore, a meta-analysis could not be performed to obtain a relevant and precise conclusion. Given the heterogeneity and shortcomings, this study should be interpreted carefully.

In conclusion, BMI ≥ 25 kg/m^2^ was a significant predictor of ARD, and hypofractionation was consistently protective. Depending on country of study, study design, and toxicity scale used, breast volume, smoking habit, diabetes, and sequential boost and bolus use were also predictive of ARD. On the basis of this study, doctors could predict patients with breast cancer at high risk of ARD at the outset of treatment options, adjust treatment plans, and take necessary precautions. In the future, more accurate predictions, such as genetic markers, are expected.

## Data Availability Statement

The raw data supporting the conclusions of this article will be made available by the authors, without undue reservation.

## Author Contributions

The authors’ responsibilities were as follows: YX and JC contributed to the conception and design of the research. YX and QW extracted the data and performed the analyses. YX, QW, and TH interpreted the evidence and wrote the manuscript. RC, JW, and HC revised the article. All authors contributed to the article and approved the submitted version.

## Conflict of Interest

The authors declare that the research was conducted in the absence of any commercial or financial relationships that could be construed as a potential conflict of interest.

## Publisher’s Note

All claims expressed in this article are solely those of the authors and do not necessarily represent those of their affiliated organizations, or those of the publisher, the editors and the reviewers. Any product that may be evaluated in this article, or claim that may be made by its manufacturer, is not guaranteed or endorsed by the publisher.

## Supplementary Material

Supplementary Figure 1 Begg’s funnel plot of patients-related risk factors associated with acute radiation dermatitis. **(A)** Age; **(B)** BMI; **(C)** breast volume; **(D)** smoking.

Supplementary Figure 2 Begg’s funnel plot of treatment-related risk factors associated with acute radiation dermatitis. **(A)** boost; **(B)** hypofractionated radiotherapy (HFRT) vs conventional fractionated radiotherapy (CFRT); **(C)** chemotherapy regimen; **(D)** hormone therapy.

Supplementary Figure 3 Sensitivity analysis of the effect of patients-related risk factors associated with acute radiation dermatitis. **(A)** Age; **(B)** BMI; **(C)** breast volume; **(D)** smoking.

Supplementary Figure 4 Sensitivity analysis of the effect of treatment-related risk factors associated with acute radiation dermatitis. **(A)** boost; **(B)** hypofractionated radiotherapy (HFRT) vs conventional fractionated radiotherapy (CFRT); **(C)** chemotherapy regimen; **(D)** hormone therapy.

Supplementary Table 1 The PRISMA 2020 checklist.

Supplementary Table 2 The search strategy.

Supplementary Table 3 Common Terminology Criteria for Adverse Events (CTCAE) or The Radiation Therapy Oncology Group (RTOG) toxicity scales for acute radiation dermatitis.

Supplementary Table 4 Quality assessment of eligible studies by Newcastle-Ottawa Scale (NOS).

Supplementary Table 5 Results of individual studies: summary statistics for each group and an effect estimate.
