# Supplementary figures and images for: Risk Factors Related to Acute Radiation Dermatitis in Breast Cancer Patients After Radiotherapy: A Systematic Review and Meta-Analysis
Source: Front Oncol. 2021 Nov 29;11:738851. doi: 10.3389/fonc.2021.738851 (PMC8667470; doi:10.3389/fonc.2021.738851)

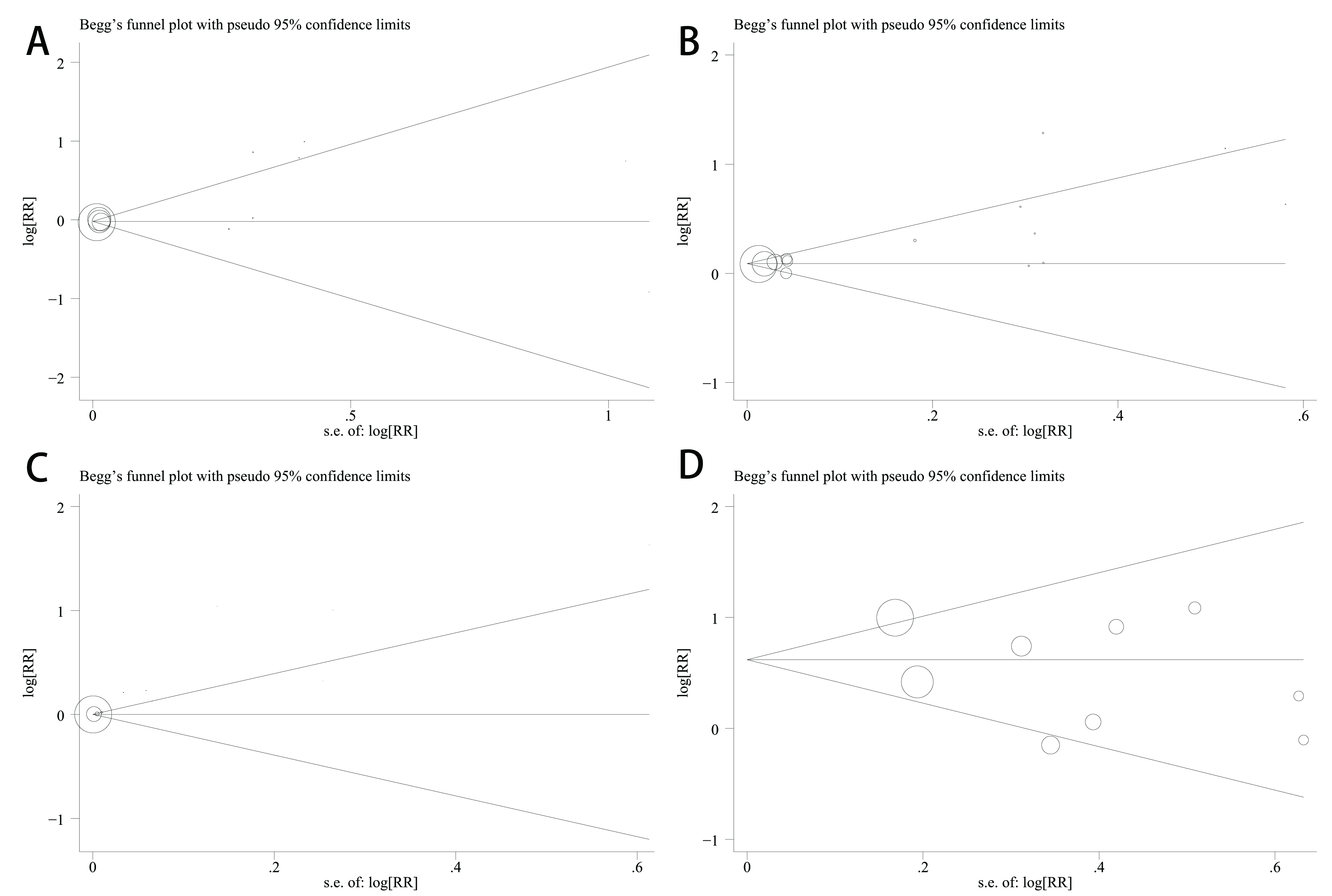

Supplement: Supplementary Figure 1 — Begg’s funnel plot of patients-related risk factors associated with acute radiation dermatitis. (A) Age; (B) BMI; (C) breast volume; (D) smoking. [file Image_1.tif]

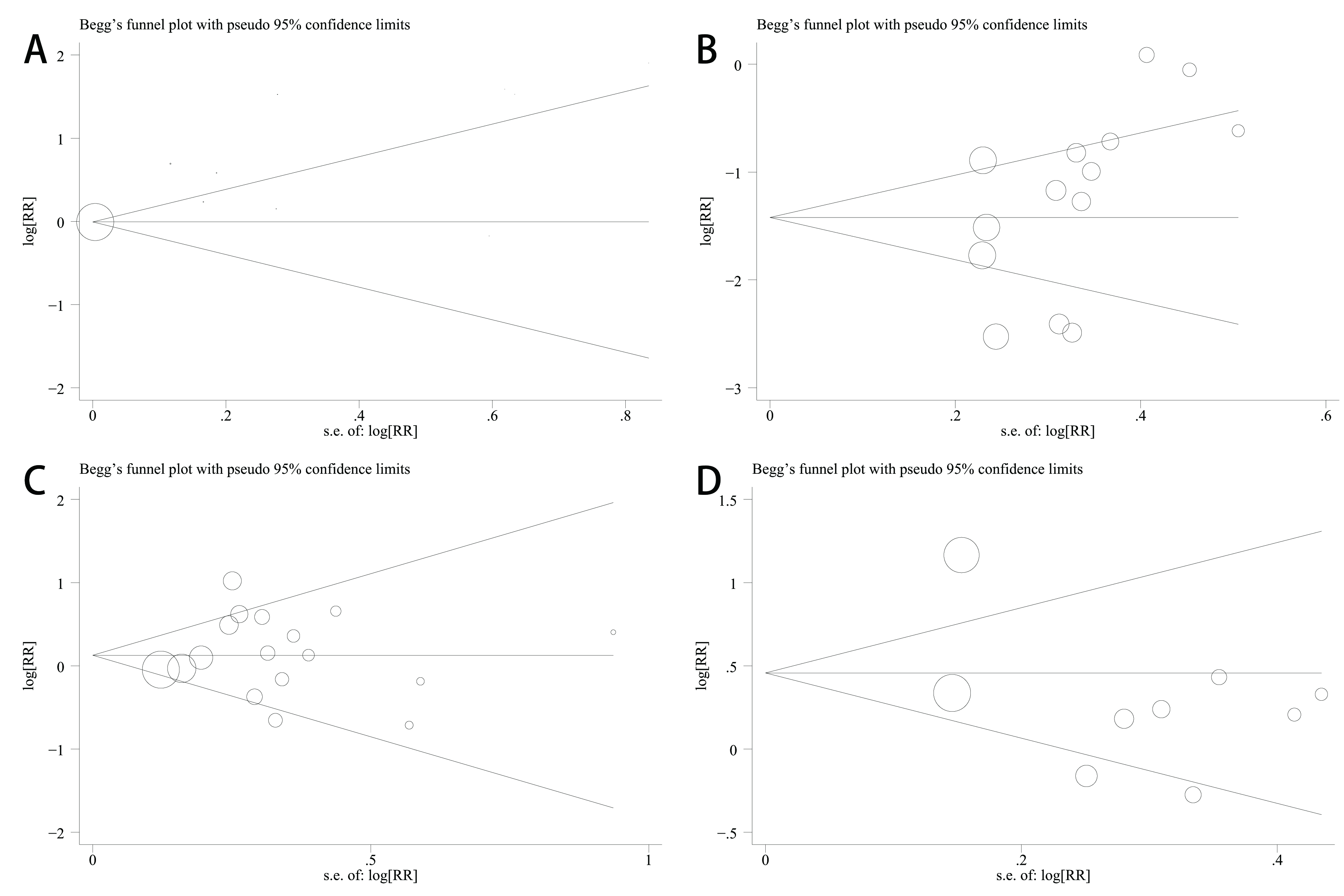

Supplement: Supplementary Figure 2 — Begg’s funnel plot of treatment-related risk factors associated with acute radiation dermatitis. (A) boost; (B) hypofractionated radiotherapy (HFRT) vs conventional fractionated radiotherapy (CFRT); (C) chemotherapy regimen; (D) hormone therapy. [file Image_2.tif]

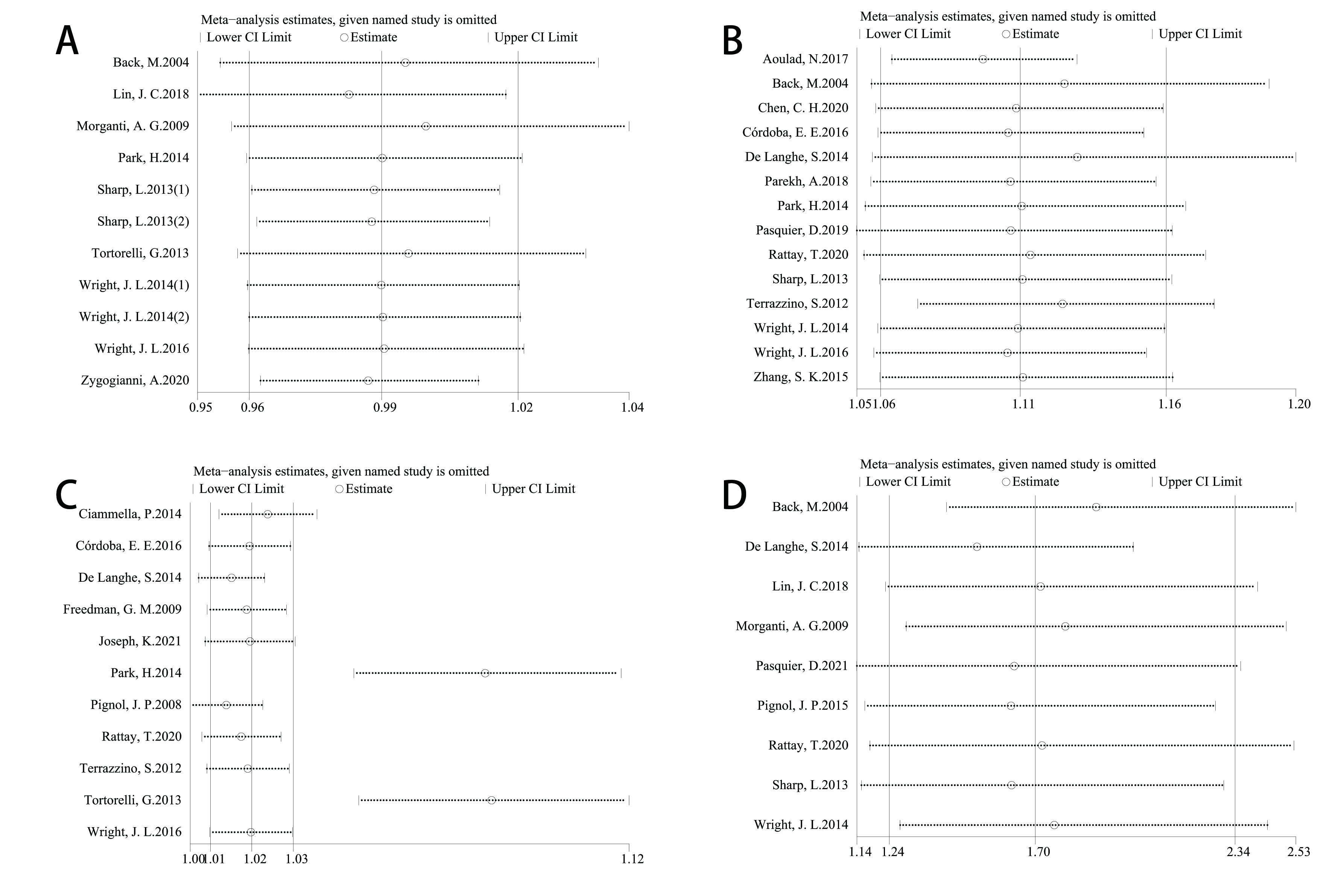

Supplement: Supplementary Figure 3 — Sensitivity analysis of the effect of patients-related risk factors associated with acute radiation dermatitis. (A) Age; (B) BMI; (C) breast volume; (D) smoking. [file Image_3.tif]

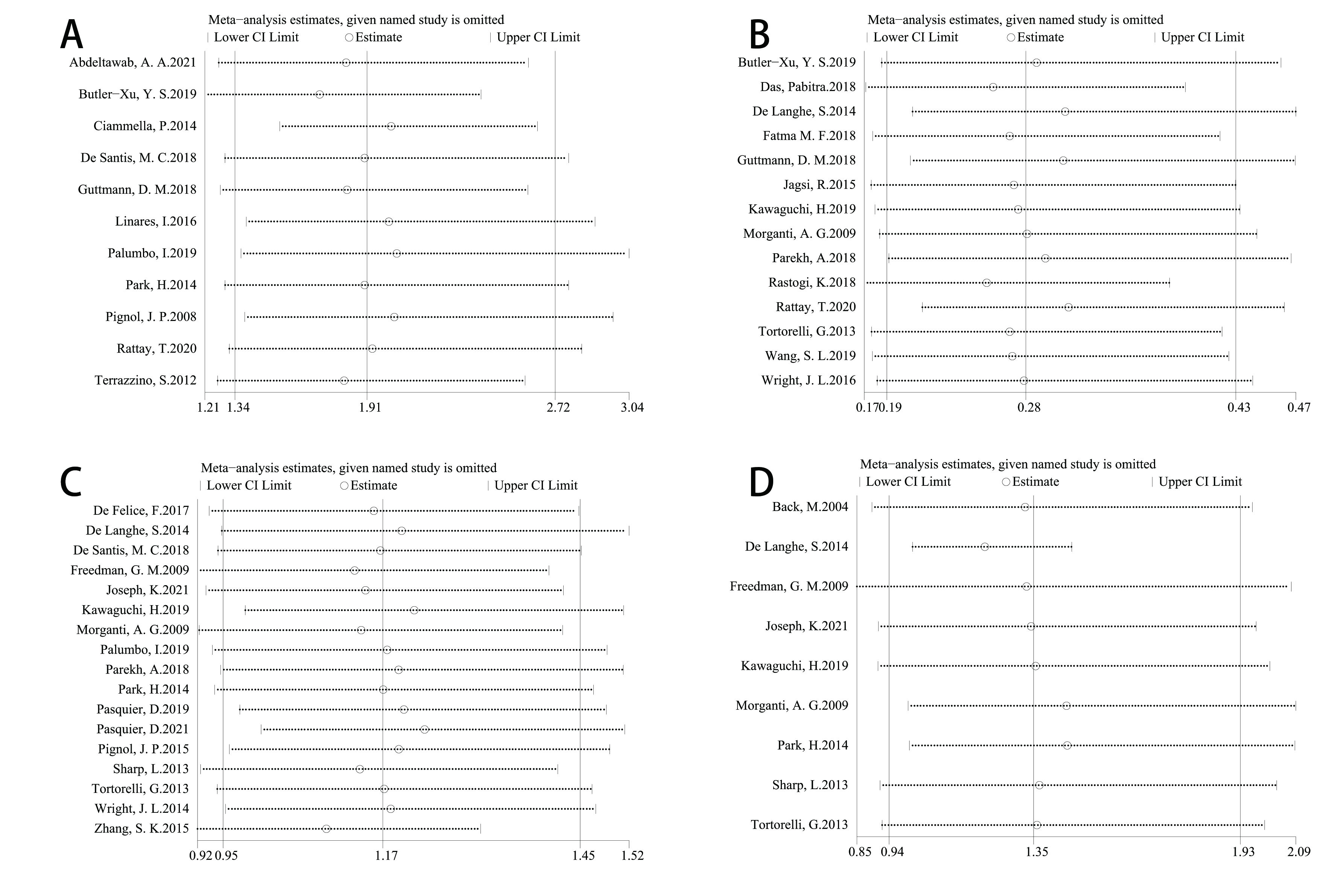

Supplement: Supplementary Figure 4 — Sensitivity analysis of the effect of treatment-related risk factors associated with acute radiation dermatitis. (A) boost; (B) hypofractionated radiotherapy (HFRT) vs conventional fractionated radiotherapy (CFRT); (C) chemotherapy regimen; (D) hormone therapy. [file Image_4.tif]
