# Supplementary material for: Risk Factors Related to Acute Radiation Dermatitis in Breast Cancer Patients After Radiotherapy: A Systematic Review and Meta-Analysis
Source: Front Oncol. 2021 Nov 29;11:738851. doi: 10.3389/fonc.2021.738851 (PMC8667470; doi:10.3389/fonc.2021.738851)
Supplement: Supplementary Table 2 — The search strategy. [file Table_2.doc]

**Supplementary Table 2. Search strategy**

| **Database** | **Search strategy** |
| --- | --- |
| **Pubmed** | #1: "[Breast Neoplasms](https://www.ncbi.nlm.nih.gov/mesh/68001943)"[Mesh]  #2: "Breast cancer"[Title/Abstract] OR "Breast carcinoma"[Title/Abstract]  #3: #1 OR #2  #4: "Radiotherapy"[Mesh]  #5: "Radiotherapy"[Title/Abstract] OR "Radiation therapy"[Title/Abstract] OR "Radiotherapy treatment"[Title/Abstract] OR "Radiation treatment"[Title/Abstract]  #6: #4 OR #5  #7: "Radiodermatitis"[Mesh]  #8: "Dermatitis"[Title/Abstract] OR "Moist desquamation"[Title/Abstract] OR "Skin toxicity"[Title/Abstract] OR "Adverse skin reactions"[Title/Abstract] OR "Radiodermatitis"[Title/Abstract] OR "Radiation dermatitis"[Title/Abstract] OR "Radiation induced skin reactions"[Title/Abstract] OR "Skin radiation toxicity" OR "Cutaneous toxicity"[Title/Abstract]  #9: ("erythema")[Title/Abstract] AND (react*[Title/Abstract] OR rash*[Title/Abstract] OR ulcer*[Title/Abstract] OR inflam*[Title/Abstract] OR red*[Title/Abstract] OR toxic*)[Title/Abstract]  #10: #7 OR #8 OR #9  #11: #3 AND #6 AND #10 |
| **Embase** | #1 'breast tumor'/exp  #2 'breast cancer':ti,ab,kw OR 'breast carcinoma':ti,ab,kw  #3 #1 OR #2  #4 'radiotherapy'/exp  #5 'radiotherapy':ti,ab,kw OR 'radiation therapy':ti,ab,kw OR 'radiotherapy treatment':ti,ab,kw OR 'radiation treatment':ti,ab,kw  #6 #4 OR #5  #7 'radiation dermatitis'/exp  #8 'dermatitis':ti,ab,kw OR 'moist desquamation':ti,ab,kw OR 'skin toxicity':ti,ab,kw OR 'adverse skin reactions':ti,ab,kw OR 'radiodermatitis':ti,ab,kw OR 'radiation dermatitis':ti,ab,kw OR 'radiation induced skin reactions':ti,ab,kw OR 'skin radiation toxicity':ti,ab,kw OR 'cutaneous toxicity':ti,ab,kw  #9 'erythema':ti,ab,kw AND (react*:ti,ab,kw OR rash*:ti,ab,kw OR ulcer*:ti,ab,kw OR inflam*:ti,ab,kw OR red*:ti,ab,kw OR toxic*:ti,ab,kw)  #10 #7 OR #8 OR #9  #11 #3 AND #6 AND #10 |
| **Cochrane Library** | #1 MeSH descriptor: [Breast Neoplasms] explode all trees  #2 ("Breast cancer" or "Breast carcinoma"):ti,ab,kw  #3 #1 or #2  #4 MeSH descriptor: [Radiotherapy] explode all trees  #5 ("Radiotherapy" or "Radiation therapy" or "Radiotherapy treatment" or "Radiation treatment"):ti,ab,kw  #6 #4 or #5  #7 MeSH descriptor: [Radiodermatitis] explode all trees  #8 ("Dermatitis" or "Moist desquamation" or "Skin toxicity" or "Adverse skin reactions" or "Radiodermatitis" or "Radiation dermatitis" or "Radiation induced skin reactions" or "Skin radiation toxicity" or "Cutaneous toxicity"):ti,ab,kw  #9 (("erythema") and (react* or rash* or ulcer* or inflam* or red* or toxic*)):ti,ab,kw  #10 #7 or #8 or #9  #11 #3 and #6 and #10 |
| **China National Knowledge Infrastructure (CNKI)** | [(主题=乳腺癌 + 乳腺肿瘤) AND (主题=放疗 + 放射治疗 + 辅助放疗 + 辅助放射治疗) AND (主题=皮炎 + 皮肤毒性 + 皮肤反应 +皮肤不良反应 )](https://kns.cnki.net/KNS8/AdvSearch?id=99&dbcode=SCDB&searchtype=gradeSearch&ishistory=1) |
| **Wanfang literature databases** | 主题:(乳腺癌 or 乳腺恶性肿瘤) and 主题:(放射治疗 or 放疗 or 辅助放疗 or辅助放射治疗) and 主题:(皮炎 or 皮肤毒性 or 皮肤反应 or 皮肤不良反应) |

All searches limited to human studies. The language was restricted to English or Chinese.
