# Supplementary material for: Risk Factors Related to Acute Radiation Dermatitis in Breast Cancer Patients After Radiotherapy: A Systematic Review and Meta-Analysis
Source: Front Oncol. 2021 Nov 29;11:738851. doi: 10.3389/fonc.2021.738851 (PMC8667470; doi:10.3389/fonc.2021.738851)
Supplement: Supplementary Table 3 — Common Terminology Criteria for Adverse Events (CTCAE) or The Radiation Therapy Oncology Group (RTOG) toxicity scales for acute radiation dermatitis. [file Table_3.doc]

| Toxicity | Grade 1 | Grade 2 | Grade 3 | Grade 4 | Grade 5 |
| --- | --- | --- | --- | --- | --- |
| RTOG | Follicular, faint or dull erythema/epilation/dry desquamation/decreased sweating | Tender or bright erythema, patchy moist desquamation/moderate edema | Confluent, moist desquamation other than skin folds, pitting edema | Ulceration, hemorrhage, necrosis |  |
| CTCAE v4.0 | Faint erythema or dry desquamation | Moderate to brisk erythema; patchy moist desquamation, mostly confined to skin folds and creases; moderate edema | Moist desquamation in areas other than skin folds and creases; bleeding induced by minor trauma or abrasion | Life-threatening consequences; skin necrosis or ulceration of full thickness dermis; spontaneous bleeding from involved site; skin graft indicated | Death |
| Definition: A finding of cutaneous inflammatory reaction occurring as a result of exposure to biologically effective levels of ionizing radiation. | | | | | |
| CTCAE v3.0 | Faint erythema or dry desquamation | Moderate to brisk erythema; patchy moist desquamation, mostly confined to skin folds and creases; moderate edema | Moist desquamation other than skin folds and creases; bleeding induced by minor trauma or abrasion | Skin necrosis or ulceration of full thickness dermis; spontaneous bleeding from involved site | Death |

**Supplementary Table 3:** Common Terminology Criteria for Adverse Events (CTCAE) or The Radiation Therapy Oncology Group (RTOG) toxicity scales for acute radiation dermatitis.
