# Supplementary material for: Risk Factors Related to Acute Radiation Dermatitis in Breast Cancer Patients After Radiotherapy: A Systematic Review and Meta-Analysis
Source: Front Oncol. 2021 Nov 29;11:738851. doi: 10.3389/fonc.2021.738851 (PMC8667470; doi:10.3389/fonc.2021.738851)
Supplement: Supplementary Table 4 — Quality assessment of eligible studies by Newcastle-Ottawa Scale (NOS). [file Table_4.doc]

| **_Supplementary Table 4_:** Quality assessment of eligible studies by Newcastle-Ottawa Scale (NOS). | | | | | | | | | | |
| --- | --- | --- | --- | --- | --- | --- | --- | --- | --- | --- |
| **Study ID** | **Study design** | **Selection** | | | | **Comparability** | **Outcome** | | | **Total NOS** |
|  |  | Representation of the exposed cohort | Selection of the non-exposed cohort | Ascertainment of exposure | Outcome of interest was not present at start of study | Comparability of cohorts on the basis of the design or analysis | Assessment of outcome | Follow-up long enough for outcomes to occur | Adequacy of follow-up of cohorts |  |
| Pasquier, D.2021 | prospective | 1 | 1 | 1 | 1 | 1 | 1 | 1 | 1 | 8 |
| Abdeltawab, A. A.2021 | prospective | 1 | 0 | 1 | 1 | 1 | 1 | 1 | 1 | 7 |
| Zygogianni, A.2020 | retrospective | 1 | 1 | 1 | 1 | 1 | 0 | 1 | 1 | 7 |
| Rattay, T.2020 | prospective | 1 | 1 | 1 | 1 | 1 | 1 | 1 | 1 | 8 |
| Chen, C. H.2020 | retrospective | 1 | 1 | 1 | 1 | 1 | 1 | 1 | 1 | 8 |
| Pasquier, D.2019 | prospective | 1 | 1 | 1 | 1 | 2 | 1 | 1 | 0 | 8 |
| Palumbo, I.2019 | prospective | 1 | 1 | 1 | 1 | 1 | 1 | 1 | 0 | 7 |
| Kawaguchi, H.2019 | prospective | 1 | 0 | 1 | 1 | 1 | 1 | 1 | 1 | 7 |
| Butler-Xu, Y. S.2019 | retrospective | 1 | 1 | 1 | 1 | 1 | 1 | 1 | 1 | 8 |
| Yap, M. L.2018 | prospective | 1 | 1 | 1 | 0 | 1 | 1 | 0 | 1 | 6 |
| Rastogi, K.2018 | prospective | 1 | 1 | 1 | 1 | 1 | 1 | 1 | 0 | 7 |
| Parekh, A.2018 | retrospective | 1 | 1 | 1 | 0 | 1 | 1 | 1 | 0 | 6 |
| Lin, J. C.2018 | retrospective | 1 | 0 | 1 | 1 | 1 | 1 | 1 | 1 | 7 |
| Guttmann, D. M.2018 | retrospective | 1 | 1 | 1 | 0 | 1 | 1 | 1 | 1 | 7 |
| De Santis, M. C.2018 | prospective | 1 | 1 | 1 | 0 | 1 | 0 | 1 | 1 | 6 |
| Das, Pabitra.2018 | prospective | 1 | 1 | 1 | 0 | 1 | 1 | 0 | 1 | 6 |
| Fatma M. F.2018 | retrospective | 1 | 1 | 1 | 1 | 1 | 1 | 1 | 0 | 7 |
| De Felice, F.2017 | prospective | 1 | 1 | 1 | 1 | 1 | 1 | 1 | 0 | 7 |
| Aoulad, N.2017 | retrospective | 1 | 1 | 1 | 1 | 1 | 1 | 0 | 1 | 7 |
| Wright, J. L.2016 | prospective | 1 | 1 | 1 | 1 | 1 | 1 | 1 | 1 | 8 |
| Linares, I.2016 | prospective | 1 | 1 | 1 | 1 | 1 | 1 | 0 | 1 | 7 |
| Córdoba, E. E.2016 | prospective | 1 | 1 | 0 | 1 | 0 | 1 | 1 | 1 | 6 |
| Zhang, S. K.2015 | prospective | 1 | 1 | 1 | 1 | 1 | 1 | 1 | 0 | 7 |
| Pignol, J. P.2015 | prospective | 1 | 1 | 1 | 1 | 1 | 1 | 0 | 1 | 7 |
| Jagsi, R.2015 | prospective | 1 | 1 | 1 | 1 | 1 | 1 | 1 | 1 | 8 |
| Wright, J. L.2014 | prospective | 1 | 1 | 1 | 1 | 1 | 1 | 1 | 1 | 8 |
| Park, H.2014 | prospective | 1 | 1 | 1 | 1 | 1 | 1 | 0 | 1 | 7 |
| De Langhe, S.2014 | prospective | 1 | 1 | 1 | 1 | 1 | 1 | 1 | 0 | 7 |
| Ciammella, P.2014 | prospective | 1 | 1 | 1 | 1 | 1 | 1 | 1 | 1 | 8 |
| Tortorelli, G.2013 | retrospective | 1 | 1 | 1 | 1 | 1 | 1 | 0 | 1 | 7 |
| Sharp, L.2013 | prospective | 1 | 1 | 1 | 1 | 1 | 1 | 0 | 1 | 7 |
| Terrazzino, S.2012 | prospective | 1 | 1 | 1 | 1 | 0 | 1 | 1 | 1 | 7 |
| Freedman, G. M.2009 | retrospective | 1 | 1 | 1 | 1 | 1 | 1 | 0 | 1 | 7 |
| Morganti, A. G.2009 | prospective | 1 | 1 | 1 | 1 | 1 | 1 | 1 | 0 | 7 |
| Back, M.2004 | prospective | 1 | 0 | 1 | 1 | 1 | 1 | 0 | 1 | 6 |
