# Supplementary material for: Risk Factors Related to Acute Radiation Dermatitis in Breast Cancer Patients After Radiotherapy: A Systematic Review and Meta-Analysis
Source: Front Oncol. 2021 Nov 29;11:738851. doi: 10.3389/fonc.2021.738851 (PMC8667470; doi:10.3389/fonc.2021.738851)
Supplement: Supplementary Table 5 — Results of individual studies: summary statistics for each group and an effect estimate. [file Table_5.doc]

| **Supplementary Table 5:** Results of individual studies: summary statistics for each group and an effect estimate. | | | | |
| --- | --- | --- | --- | --- |
| **Study ID** | **Risk factors** | **RR** | **95% CI** | |
| Abdeltawab, A. A.2021 | Boost:Yes vs. No | 4.61 | 1.33 | 15.93 |
|  | Trastuzumab: Yes vs. No | 4.53 | 1.27 | 16.14 |
| Aoulad, N.2017 | BMI | 3.61 | 1.93 | 6.74 |
| Back, M.2004 | Age | 0.98 | 0.96 | 1.01 |
|  | BMI | 1.09 | 1.05 | 1.13 |
|  | Hormone treatment：Yes vs. No | 1.54 | 0.77 | 3.09 |
|  | Nodal irradiation: Yes vs. No | 1.27 | 0.66 | 2.47 |
|  | Smoking: Yes vs. No | 0.86 | 0.44 | 1.7 |
| Butler-Xu, Y. S.2019 | Boost:Yes vs. No | 4.6 | 2.67 | 7.92 |
|  | Treatment group:HFRT VS. CFRT | 0.22 | 0.14 | 0.35 |
| Chen, C. H.2020 | BMI | 1.44 | 0.78 | 2.63 |
|  | Nodal irradiation: Yes vs. No | 1.23 | 0.91 | 1.67 |
| Ciammella, P.2014 | Boost:Yes vs. No | 0.9909 | 0.984 | 0.998 |
|  | Breast volume: large vs. small | 1.0061 | 0.997 | 1.016 |
| Córdoba, E. E.2016 | BMI | 3.14 | 1.13 | 8.53 |
|  | Breast volume: large vs. small | 5.11 | 1.54 | 17.04 |
| Das, Pabitra.2018 | Treatment group:HFRT VS. CFRT | 0.95 | 0.39 | 2.3 |
| De Felice, F.2017 | Chemotherapy：Yes vs. No | 1.43 | 0.68 | 2.8 |
| De Langhe, S.2014 | BMI | 1.088 | 1.062 | 1.115 |
|  | Breast volume: large vs. small | 2.833 | 2.166 | 3.705 |
|  | Chemotherapy：Yes vs. No | 0.954 | 0.75 | 1.213 |
|  | Hormone treatment：Yes vs. No | 3.207 | 2.375 | 4.33 |
|  | Smoking: Yes vs. No | 2.711 | 1.95 | 3.77 |
|  | Trastuzumab: Yes vs. No | 0.177 | 0.113 | 0.277 |
|  | Treatment group:HFRT VS. CFRT | 0.083 | 0.044 | 0.158 |
| De Santis, M. C.2018 | Boost:Yes vs. No | 2 | 1.59 | 2.51 |
|  | Chemotherapy：Yes vs. No | 1.5 | 0.24 | 9.42 |
|  | Trastuzumab: Yes vs. No | 0.4 | 0.27 | 0.57 |
| Fatma M. F.2018 | Treatment group:HFRT VS. CFRT | 0.54 | 0.2 | 1.45 |
| Freedman, G. M.2009 | Breast volume: large vs. small | 2.73 | 1.63 | 4.6 |
|  | Chemotherapy：Yes vs. No | 1.86 | 1.11 | 3.12 |
|  | Hormone treatment：Yes vs. No | 1.4 | 1.05 | 1.86 |
| Guttmann, D. M.2018 | Boost:Yes vs. No | 6.7 | 1.3 | 34.28 |
|  | Treatment group:HFRT VS. CFRT | 0.09 | 0.05 | 0.17 |
| Jagsi, R.2015 | Treatment group:HFRT VS. CFRT | 0.41 | 0.26 | 0.64 |
| Joseph, K.2021 | Breast volume: large vs. small | 1.02 | 1.001 | 1.04 |
|  | Chemotherapy：Yes vs. No | 1.93 | 0.82 | 4.55 |
|  | Hormone treatment：Yes vs. No | 1.39 | 0.59 | 3.24 |
| Kawaguchi, H.2019 | Chemotherapy：Yes vs. No | 0.69 | 0.39 | 1.22 |
|  | Hormone treatment：Yes vs. No | 1.27 | 0.69 | 2.32 |
|  | Treatment group:HFRT VS. CFRT | 0.37 | 0.19 | 0.74 |
| Lin, J. C.2018 | Age | 1.01 | 0.99 | 1.04 |
|  | Smoking: Yes vs. No | 1.34 | 0.39 | 4.55 |
| Linares, I.2016 | Boost:Yes vs. No | 0.84 | 0.26 | 2.68 |
| Morganti, A. G.2009 | Age | 0.97 | 0.96 | 0.99 |
|  | Chemotherapy：Yes vs. No | 1.63 | 1.01 | 2.64 |
|  | Hypertension:Yes VS. No | 1.01 | 0.64 | 1.62 |
|  | Diabetes：Yes VS. No | 1.77 | 0.77 | 4.13 |
|  | Hormone treatment：Yes vs. No | 0.85 | 0.52 | 1.39 |
|  | Smoking: Yes vs. No | 1.06 | 0.49 | 2.29 |
|  | Treatment group:HFRT VS. CFRT | 0.28 | 0.15 | 0.56 |
| Palumbo, I.2019 | Boost:Yes vs. No | 1.262 | 0.911 | 1.747 |
|  | Chemotherapy：Yes vs. No | 1.105 | 0.754 | 1.621 |
| Parekh, A.2018 | BMI | 1.35 | 0.95 | 1.93 |
|  | Chemotherapy：Yes vs. No | 0.97 | 0.71 | 1.33 |
|  | Nodal irradiation: Yes vs. No | 2.4 | 1.62 | 3.57 |
|  | Race:Black | 0.75 | 0.56 | 1.01 |
|  | Treatment group:HFRT VS. CFRT | 0.17 | 0.11 | 0.27 |
| Park, H.2014 | Age | 1.023 | 0.557 | 1.879 |
|  | BMI | 1.118 | 1.026 | 1.217 |
|  | Boost:Yes vs. No | 2 | 1.59 | 2.51 |
|  | Breast volume: large vs. small | 1.004 | 1.002 | 1.007 |
|  | Chemotherapy：Yes vs. No | 1.164 | 0.628 | 2.156 |
|  | Hypertension:Yes VS. No | 1.15 | 0.39 | 3.44 |
|  | Diabetes：Yes VS. No | 1.89 | 0.51 | 6.94 |
|  | Hormone treatment：Yes vs. No | 0.759 | 0.394 | 1.461 |
| Pasquier, D.2019 | BMI | 1.14 | 1.05 | 1.24 |
|  | Chemotherapy：Yes vs. No | 0.49 | 0.16 | 1.49 |
| Pasquier, D.2021 | Chemotherapy：Yes vs. No | 0.52 | 0.27 | 0.98 |
|  | Smoking: Yes vs. No | 2.1 | 1.14 | 3.87 |
| Pignol, J. P.2008 | Boost:Yes vs. No | 1.162 | 0.677 | 1.993 |
|  | Breast volume: large vs. small | 1.236 | 1.157 | 1.321 |
| Pignol, J. P.2015 | Chemotherapy：Yes vs. No | 0.85 | 0.44 | 1.67 |
|  | Smoking: Yes vs. No | 2.96 | 1.09 | 8.01 |
|  | Bolus: yes vs. no | 3.68 | 2.05 | 6.26 |
| Rastogi, K.2018 | Treatment group:HFRT VS. CFRT | 1.09 | 0.49 | 2.41 |
| Rattay, T.2020 | BMI | 1.11 | 1.05 | 1.18 |
|  | Boost:Yes vs. No | 1.79 | 1.24 | 2.57 |
|  | Breast volume: large vs. small | 1.26 | 1.12 | 1.41 |
|  | Smoking: Yes vs. No | 1.52 | 1.04 | 2.22 |
|  | Treatment group:HFRT VS. CFRT | 0.08 | 0.05 | 0.13 |
| Sharp, L.2013 | BMI | 1.1 | 0.6 | 2.1 |
|  | Chemotherapy：Yes vs. No | 1.8 | 1 | 3.3 |
|  | Hormone treatment：Yes vs. No | 1.2 | 0.7 | 2.1 |
|  | Smoking: Yes vs. No | 2.5 | 1.1 | 5.7 |
| Sharp, L.2013(1) | Age | 2.2 | 1 | 4.8 |
| Sharp, L.2013(2) | Age | 2.7 | 1.2 | 6 |
| Terrazzino, S.2012 | BMI | 1.002 | 0.923 | 1.089 |
|  | Boost:Yes vs. No | 4.902 | 1.458 | 16.483 |
|  | Breast volume: large vs. small | 1.138 | 1.001 | 1.293 |
| Tortorelli, G.2013 | Age | 0.973 | 0.943 | 1.005 |
|  | Breast volume: large vs. small | 1.001 | 1 | 1.002 |
|  | Chemotherapy：Yes vs. No | 1.137 | 0.531 | 2.433 |
|  | Hormone treatment：Yes vs. No | 1.23 | 0.547 | 2.765 |
|  | Treatment group:HFRT VS. CFRT | 0.489 | 0.238 | 1.004 |
| Wang, S. L.2019 | Treatment group:HFRT VS. CFRT | 0.44 | 0.23 | 0.84 |
| Wright, J. L.2014 | BMI | 1.88 | 0.6 | 5.84 |
|  | Chemotherapy：Yes vs. No | 0.83 | 0.26 | 2.62 |
|  | Race:AA vs. Non-AA | 1.57 | 0.14 | 17.11 |
|  | Race：Hispanic vs. non-Hispanic | 0.69 | 0.08 | 6.04 |
|  | Smoking: Yes vs. No | 0.9 | 0.26 | 3.10 |
| Wright, J. L.2014(1) | Age | 2.11 | 0.28 | 16.07 |
| Wright, J. L.2014(2) | Age | 0.4 | 0.05 | 3.43 |
| Wright, J. L.2016 | Age | 0.89 | 0.53 | 1.49 |
|  | BMI | 1.84 | 1.03 | 3.27 |
|  | Breast volume: large vs. small | 1.38 | 0.84 | 2.27 |
|  | Race:AA vs. Non-AA | 1.01 | 0.58 | 1.76 |
|  | Treatment group:HFRT VS. CFRT | 0.31 | 0.17 | 0.57 |
| Yap, M. L.2018 | Bolus: yes vs. no | 2.25 | 1.2 | 4.23 |
| Zhang, S. K.2015 | BMI | 1.07 | 0.59 | 1.94 |
|  | Chemotherapy：Yes vs. No | 2.78 | 1.7 | 4.55 |
|  | Diabetes：Yes VS. No | 2.44 | 1.56 | 3.82 |
| Zygogianni, A.2020 | Age | 2.36 | 1.11 | 3.75 |

HFRT: hypofractionated radiotherapy; CFRT: conventional fractionated radiotherapy; AA: African American; RR: relative risk; 95% CI: 95% confidence intervals
